# Effect of Osteoperiosteal Decortication With Bone Grafting on Healing of Femoral Fracture Nonunion

**DOI:** 10.1155/cro/9993269

**Published:** 2026-07-19

**Authors:** Mohammed M. Elgack, Ahmed Mohamed Sonbol, Farid N. Kassab, Mohamed Awad A. Mohamed, Rima Yassin, Elsayed Shaheen, Hassan Sirajaldeen Alhassan Ali

**Affiliations:** ^1^ Orthopaedic Department, King′s College Hospital London, Jeddah, Saudi Arabia; ^2^ Orthopedic Department, Dr. Soliman Fakeeh Hospital, Fakeeh Care Group, Jeddah, Saudi Arabia, dsfh.med.sa; ^3^ Orthopedic Department, International Medical Center, Jeddah, Saudi Arabia, imc.med.sa; ^4^ Orthopedic Department, Al-Alzhar University, Nasr City, Egypt

**Keywords:** bone graft, femur fracture, nonunion, osteoperiosteal decortication

## Abstract

Femoral fractures are common injuries, typically demonstrating favorable outcomes with appropriate treatment. However, nonunion may occur due to several reasons. Osteoperiosteal decortication with bone grafting, initially described by Judet in 1969, has been recognized as a safe and effective method for managing nonunions. In this study, we aimed to determine the efficacy of this technique based on our experience in treating femoral nonunion. This case series included patients with femoral fracture nonunion who were treated with osteoperiosteal decortication performed by a single surgeon between April 2011 and January 2020. Data was collected from patients′ medical records and hospital documents. The primary outcome measure was the attainment of clinical and radiographic unions during the follow‐up period. The study included eight patients, including seven patients with diaphyseal fractures. Union was successfully achieved in all the patients. The mean time to union was approximately 6.13 ± 2 months. Five patients resumed full weight‐bearing activities before 6 months. No additional procedures were required for any patient. In conclusion, osteoperiosteal decortication with bone grafting is an effective treatment approach for managing nonunions with promising success rates. The outcomes were satisfactory, as evaluated through clinical and radiological examinations.

## 1. Introduction

Femoral fractures are frequent traumatic injuries that necessitate hospitalization and surgical intervention [1]. The etiology of these fractures varies depending on the specific type. Peritrochanteric and distal femoral fractures are commonly attributed to falls and osteoporosis in older individuals [2]. Conversely, shaft fractures are typically associated with motor vehicle accidents in younger population [3]. Despite the relatively low nonunion rate of 2% achieved with reamed intramedullary nailing for femoral diaphyseal fractures, nonunions can result in substantial morbidity and cost implications [4, 5]. This incidence increases in osteoporotic fractures, as demonstrated by Parker et al. [6] who reported a nonunion rate of 19% in all patients with proximal femoral fractures, with a higher prevalence among older patients, particularly those aged > 70 years (24.9%). Femoral nonunion is typically characterized clinically by persistent pain and pseudoarthrosis, and radiographically by the absence of union within 6–12 months after fixation, lack of healing progress over the last 3 months, or evident implant failure [7]. Nonunion of fractures arises from a complex interplay of various factors that can be broadly classified into local, biological, and systemic causes [8–10]. Systemic factors include comorbidities, such as diabetes, hypertension, thyroid disorders, and osteoporosis, as well as age and certain social habits, such as smoking and excessive use of nonsteroidal anti‐inflammatory drugs. Local causes include fracture stability, inadequate bone contact, vascularity of the affected area, and presence of infection.

Several surgical interventions and adjuncts have been developed, leading to significant advancements and enhanced outcomes, including internal and external fixation, bone grafting, bone transport utilizing distraction osteomodulation, and decortication techniques such as the pioneering method introduced by Robert Judet in 1962 [11]. In this particular case series, we present our experience with osteoperiosteal decortication, which, to the best of our knowledge, represents the first study in Saudi Arabia to adopt this method for the management of femoral nonunion.

## 2. Case Presentations

**Figure 1:**
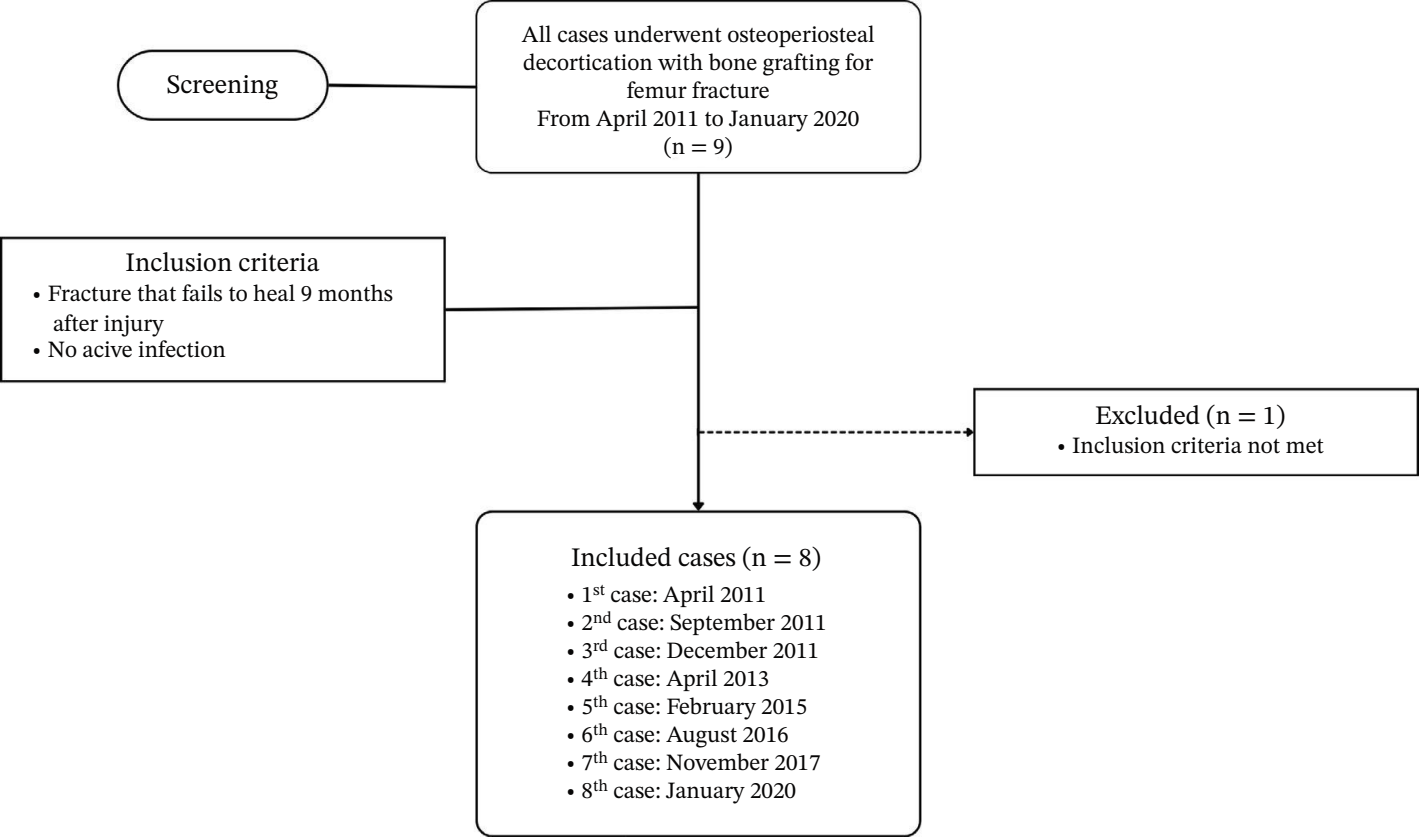
Description of the study case series.

This study followed the principles of the Helsinki Declaration and was approved by the institutional review board of the International Medical Center Hospital (No. 2023‐08‐224). The requirement for informed consent was waived considering the retrospective nature of the study. A retrospective review was conducted including patients with femoral fracture nonunion who underwent osteoperiosteal decortication by a single surgeon between April 2011 and January 2020. The inclusion criteria encompassed patients whose data were obtained from intraoperative notes, hospital records, and radiology images. Nonunion was defined according to established AO principles [12] as a fracture that fails to show evidence of healing between 6 and 8 months after injury, or, in other descriptions, as persistent lack of union after 9 months despite appropriate therapeutic measures. The steps for including subjects in this consecutive series are described in Figure 1.

Nonunions were further classified following Weber and Cech [13] into atrophic, hypertrophic, or oligotrophic types: atrophic nonunion reflects poor vascularity with limited osteogenic potential and minimal or absent callus around a fibrous gap; hypertrophic nonunion is associated with inadequate mechanical stability in the presence of good vascularity and is characterized radiographically by abundant callus with a persistent fracture line; and oligotrophic nonunion represents a biologically viable but mechanically unfavorable state, in which little or no callus forms despite adequate blood supply, typically after major displacement or inaccurate fixation, with radiographs showing absent callus and progressive bone end resorption over 8–12 weeks. Septic nonunion with active infection was excluded in all patients preoperatively on the basis of history, clinical examination, and laboratory investigations, and intraoperatively through microbiological cultures.

**Figure 2:**
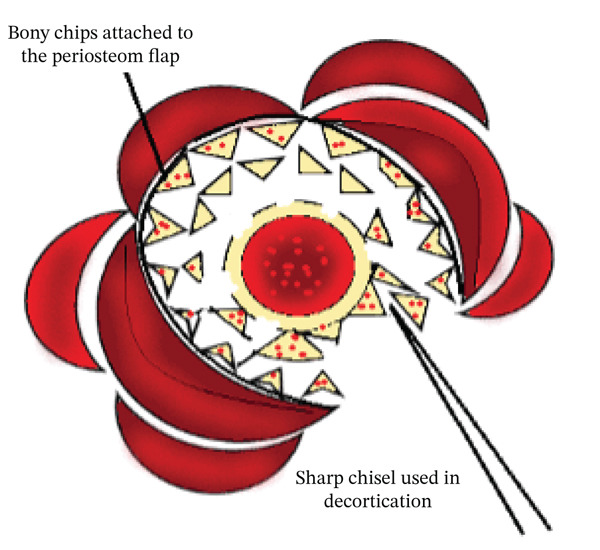
Illustration showing musculo‐osteoperiosteal decortication.

**Figure 3:**
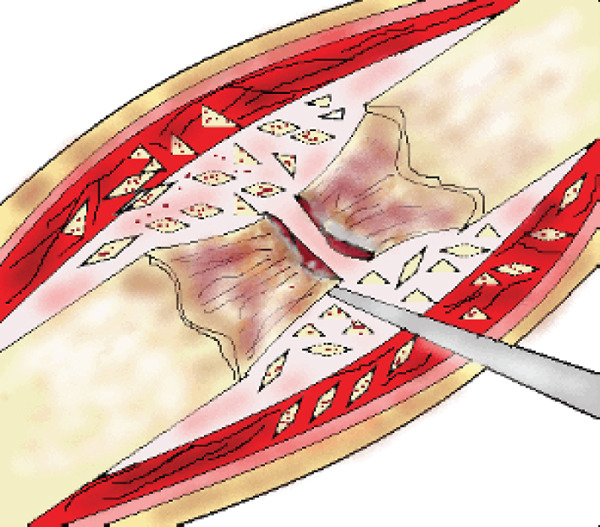
Illustration showing decorticated area around the nonunion site.

**Figure 4:**
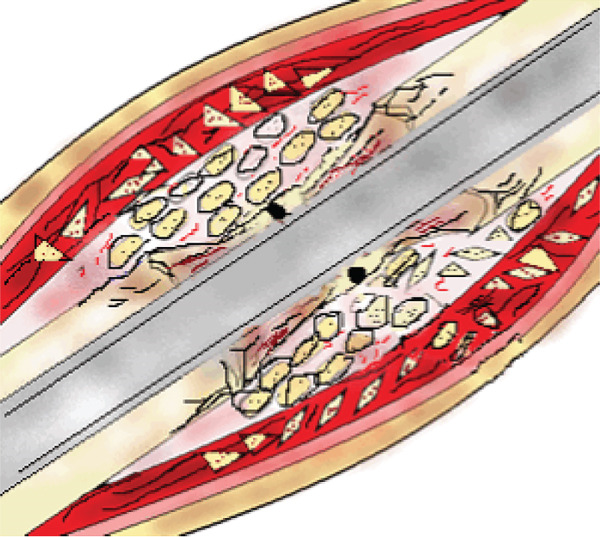
Illustration demonstrating fracture site fixed with intramedullary nail and encased with auto‐allograft.

The surgical technique employed in this study was similar to Judet′s approach. The procedure began with a single incision that extended through the muscle layers, reaching the bone and fracture sites without separating the tissue layers. Subsequently, the previous implants were removed, and a cautery device was used superficially to mark the intended decortication area. A sharp chisel was then used to perform decortication, generating small bone chips that remained attached to the periosteum and muscle, thereby preserving the blood supply (Figures 2 and 3). In cases with local comminution or butterfly fragments, larger fragments were crushed into smaller chips while preserving their soft‐tissue attachments. The osteoperiosteal decortication flap was fashioned by elevating cortical chips approximately 1–3 mm thick, extending 5–10 cm proximally and distally from the nonunion site and encompassing roughly 60%–75% of the bone circumference. The underlying bone is subsequently debrided or osteotomized as required. Subsequently, intramedullary reaming was performed with a hand reamer to reopen the medullary canal and refresh the sclerotic fracture edges. In cases with prior plate fixation, reaming was extended proximally up to the level just proximal to the most proximal screw of the previous construct. Following this, reduction and fixation were achieved using newly introduced fixation implants, such as plates or nailing systems. Reduction was assessed intraoperatively using fluoroscopy to evaluate coronal, sagittal, and axial alignment, as well as rotation, and was considered acceptable when the mechanical axis was restored without visible malalignment or rotational discrepancy. Bone contact at the nonunion site was estimated under direct visualization and fluoroscopy, with a surgical goal of at least 70%–80% cortical contact; in this series all cases achieved ≥ 50% contact. The fixation strategy was tailored to fracture site, morphology, and bone quality. Intramedullary nailing was preferred for diaphyseal shaft fractures, locked plates were used for lower‐third diaphyseal and supracondylar fractures, and 95° blade plates were selected for subtrochanteric fractures. In the final stage of the procedure, cancellous bone graft was packed circumferentially into the decorticated region around the nonunion site, both beneath the osteoperiosteal flap and along the fracture gap. The graft consisted of either autologous iliac crest bone, synthetic allograft, or combined, with the choice of material applied similarly in cases treated with plates or intramedullary nails. The volume of graft was adjusted to the size of the defect, typically ranging from approximately 5 cc in small gaps to 30 cc of bone‐chip allograft in larger defects. Before closure, the periosteal–muscular (osteoperiosteal) envelope was meticulously sutured to secure the graft in intimate contact with the fracture site and underlying implant, thereby maintaining graft containment and maximizing the graft–host bone interface (Figure 4). Radiographic union was defined as bridging callus across at least three of four cortices on standard orthogonal radiographic views. Follow‐up imaging was obtained monthly for all patients until radiographic evidence of healing was observed, then at 3‐monthly intervals for two subsequent visits, and thereafter every 6 months.

### 2.1. Case 1

**Figure 5:**
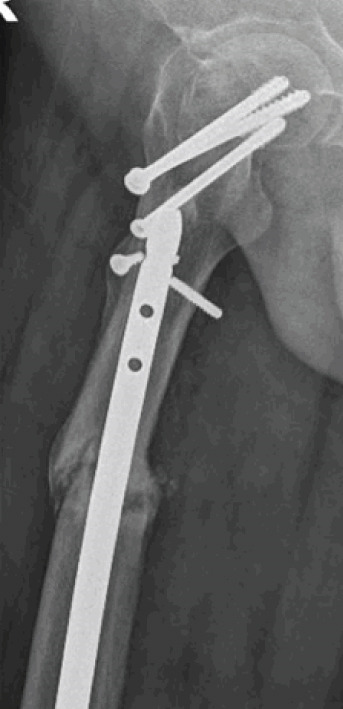
Preoperative X‐ray image showing a right femoral shaft nonunion in a 59‐year‐old male patient with a 3‐month history of a right neck of femur fracture accompanied by an ipsilateral femoral shaft fracture. The initial treatment involved cannulated screws and a retrograde femoral nail.

**Figure 6:**
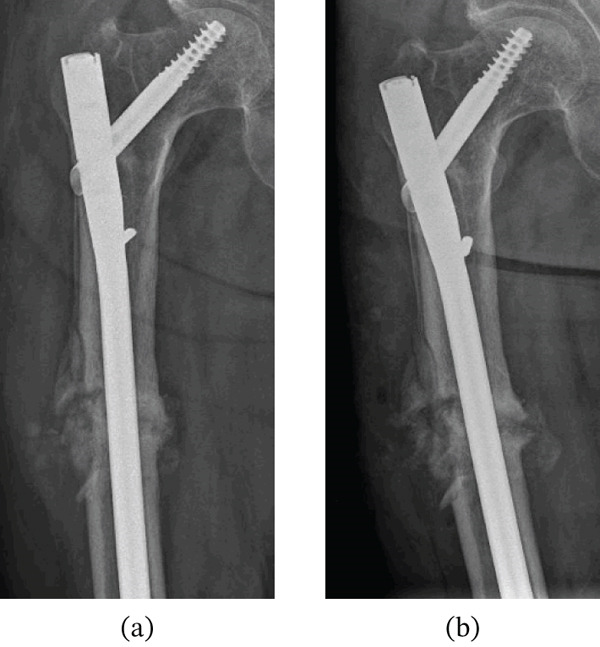
Panel (a) depicts an immediate postoperative X‐ray image following the procedure involving decortication, bone grafting, and the introduction of an antegrade cephalomedullary nail. On the other hand, Panel (b) displays a 3‐month postoperative X‐ray image showcasing the formation of callus at the site of the fracture.

**Figure 7:**
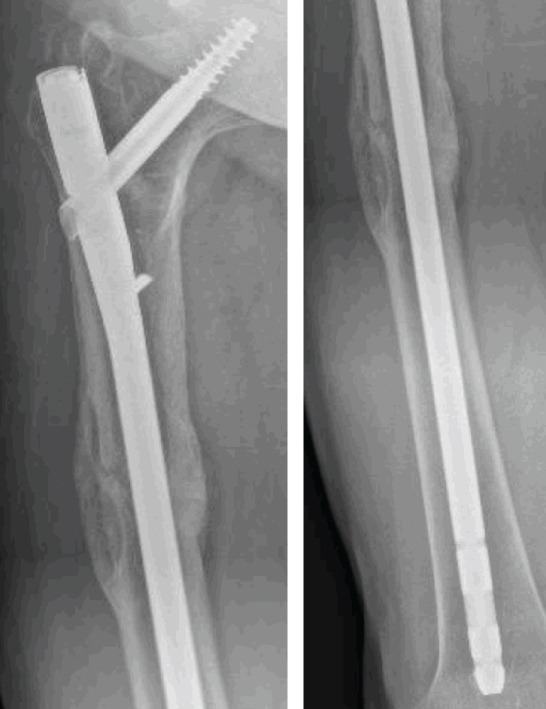
The X‐ray image taken 9 months after the osteoperiosteal decortication procedure, revealing successful fracture union.

A 59‐year‐old gentleman with a medical history significant for diabetes mellitus, hypertension, dyslipidemia, and ischemic heart disease presented with a previous history of a right neck of femur fracture and a concomitant ipsilateral upper femur shaft fracture, located just distal to the subtrochanteric area. These fractures were managed surgically with two separate fixation devices: cannulated screws for the neck fracture and a retrograde intramedullary nail for the shaft fracture. Eighteen months postfixation, while bearing weight, the patient presented to the emergency room with hip pain following minor trauma. Clinical examination revealed a well‐healed surgical incision with no signs of infection. Radiographic imaging demonstrated an atrophic nonunion with a fractured intramedullary nail (Figure 5). The previously implanted hardware was removed, and the nonunion was treated with osteoperiosteal decortication and fixation using a cephalomedullary nail, supplemented with a bone allograft (Figure 6). Sufficient radiographic union was evident at 9 months postoperatively (Figure 7).

### 2.2. Case 2

**Figure 8:**
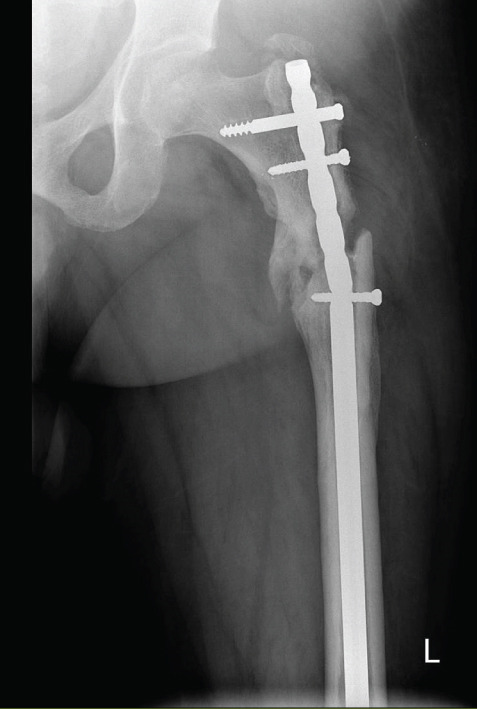
Preoperative X‐ray image of a 44‐year‐old male left femur subtrochunteric fracture nonunion evident by broken intramedullary nail.

**Figure 9:**
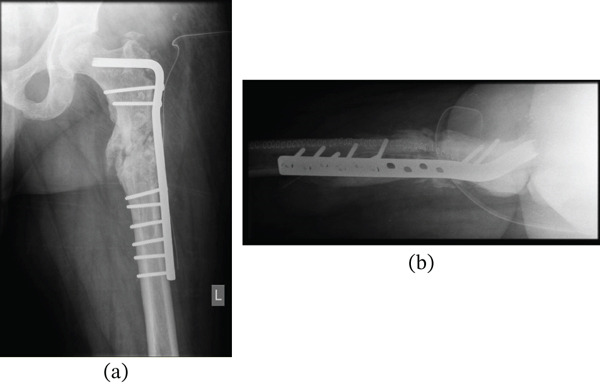
Panel (a) depicts an immediate postoperative X‐ray image following the procedure involving decortication, bone grafting by autologous bone graft and allograft, and the fixation by blade plate. Panel (b) shows the other view.

**Figure 10:**
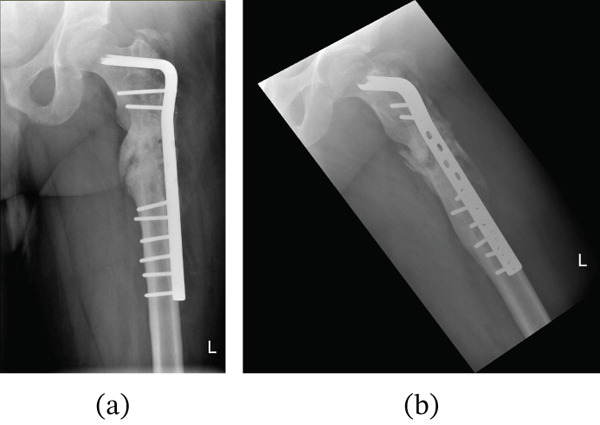
(a) The anteroposterior view 4 months postoperatively shows evidence of bone bridge and union, whereas (b) shows the lateral view.

**Figure 11:**
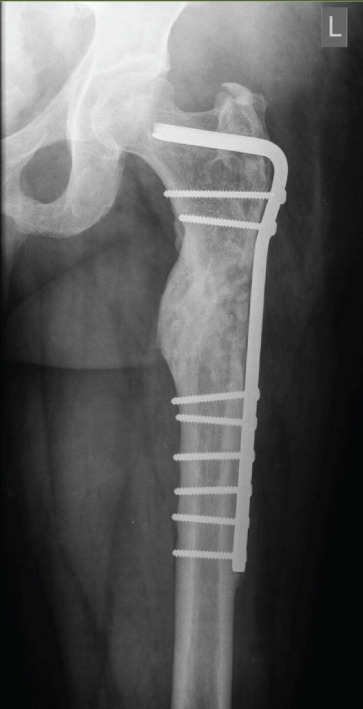
One year postoperatively showing complete healing.

A 44‐year‐old male with no significant medical comorbidities presented with a history of a traumatic subtrochanteric femur fracture. This fracture had been fixed 2 years prior with a cephalomedullary nail. He was subsequently diagnosed with a hypertrophic nonunion and presented with a fractured intramedullary nail (Figure 8). Treatment involved osteoperiosteal decortication with the addition of an iliac crest bone graft and allograft, and fixation with a 95° blade plate (Figure 9). Progressive clinical and radiographic union was observed on follow‐up imaging (Figures 10 and 11).

### 2.3. Case 3

A 19‐year‐old female presented with a history of a traumatic midshaft femur fracture, which had been fixed 1 year prior with a plate and screws at another hospital. She reported persistent pain with weight‐bearing. An infection workup yielded negative results. Radiographic imaging revealed an oligotrophic nonunion. The patient underwent osteoperiosteal decortication with an iliac crest bone graft and allograft, and fixation with an intramedullary femoral nail. After 3 months, there was insufficient evidence of healing, necessitating dynamization. One month later, good callus formation was observed on radiographs, with progressive healing noted on subsequent follow‐up examinations.

### 2.4. Case 4

A 54‐year‐old lady with a known history of osteoporosis, managed with bisphosphonates, and autoimmune hepatitis, presented with a history of a subtrochanteric atypical femur fracture sustained 2 years prior. This fracture was initially managed by open reduction and internal fixation with a blade plate. The patient presented to the emergency room with a fractured plate and an atrophic nonunion. Treatment involved osteoperiosteal decortication with autograft and allograft, and fixation with a blade plate. Progressive healing was evident on radiographic imaging, with union achieved by 5 months postoperatively.

### 2.5. Case 5

A 71‐year‐old lady with poor bone quality presented with a history of a midshaft femur fracture sustained 15 months prior. She presented with a fractured intramedullary nail and an atrophic nonunion. Initially, she underwent revision open reduction and internal fixation with a dynamic compression plate, but this failed 10 days later following a mechanical fall. The patient was taken back to surgery, where osteoperiosteal decortication was performed with the addition of allograft and iliac crest autograft, and fixation with an intramedullary nail. Radiographic evidence of fracture union was observed by 6 months postoperatively.

### 2.6. Case 6

A 58‐year‐old lady with a medical history of diabetes mellitus, hypertension, dyslipidemia, obstructive sleep apnea, and asthma presented with a periprosthetic fracture with an intact total knee replacement femoral implant, which had been fixed 1 year prior with a distal femoral locked plate. She presented with persistent mechanical pain, and radiographic imaging revealed an atrophic nonunion. Treatment involved osteoperiosteal decortication with autograft and allograft and fixation with another distal femoral locked plate. Radiographic union was evident by 5 months postoperatively.

### 2.7. Case 7

A 59‐year‐old lady presented with an atrophic nonunion of a supracondylar femur fracture, which had been fixed 18 months prior with a distal femoral locked plate. Her medical history included diabetes mellitus, hypertension, dyslipidemia, vitamin D deficiency, anemia, and peripheral vascular disease status post ipsilateral angioplasty. Treatment involved osteoperiosteal decortication with autograft and allograft, and fixation with another distal femoral locked plate. Fracture union was evident by 9 months.

### 2.8. Case 8

A 44‐year‐old lady presented with a midshaft femur fracture that had been treated 9 months previously at another hospital with open reduction and internal fixation using a distal femur locked plate. The case history indicated a previous infection at the fracture site, which had been successfully addressed with antibiotic treatment. At the time of presentation, there were no clinical indications of ongoing infection before or during surgery, and culture findings were normal. Therefore, osteoperiosteal decortication was performed with allograft alone and fixation with a distal femoral locked plate. Clinical and radiographic union was evident by 7 months postoperatively.

The primary outcome measure was the attainment of fracture union, defined as the ability to bear weight without pain, supported by radiographic evidence of bridging calluses in both the sagittal and coronal planes. Radiographs were reviewed by a blinded and independent reviewer. The time required for union was evaluated during the follow‐up period.

This consecutive case series included eight patients (six female and two male), aged 19–71 years, who underwent treatment with the osteoperiosteal decortication technique. All patients had previously undergone initial fixation surgery within 6–8 months prior to subsequent surgical intervention, and none exhibited clinical or radiological indications of fracture union.

The mean age of the patients was 51 years. One patient was on bisphosphonate treatment and had an atypical fracture. None of our cohort reported a history of smoking or use of steroids or nonsteroidal anti‐inflammatory medications. All patients had normal levels of serum calcium.

Fracture union was successfully achieved in all patients, as confirmed through clinical assessments and radiological examinations during their follow up. Five of the patients achieved union within or before 6 months, enabling them to resume full weight‐bearing activities. The remaining patients experienced complete fracture healing after 6 months, with three achieving union at the 9‐month mark. The mean time to union was ±SD: 6.13 ± 2.0 months.

The procedural steps for all patients involved the removal of previous implants, which varied in types, including different types of plates and nails. Among the patients, five had fixed plates and three had intramedullary nails. These implants were removed, and the fracture site was decorticated before the placement of new implants. In five patients, a new plate was affixed to the fracture site, whereas the remaining three patients underwent intramedullary nailing.

The final step of the procedure involved bone grafting. Six patients received a combination of autografts, primarily sourced from the iliac crest, and allografts in the form of synthetic bone chips and putty. The remaining two patients exclusively received allografts during the procedure. Summary of the cases is presented in Table 1.

**Table 1:** Patients′ detailed data.

Patient	Sex	Age (years)	Comorbidities	Site	Bone quality	Number and type of previous surgeries	Nonunion type (atrophic, hypertrophic, oligotrophic)	Mechanism of implant failure (biological/mechanical)	Previous implant	New implant used	Graft used	Time to union (months)	NUSS score
1	Male	59	DM, HTN, DL, IHD	Shaft diaphyseal	Moderate	1, primary fracture fixation	Atrophic	Biological	Retrograde nail	Cephalomedullary nail	Allograft	9	28
2	Male	44	None	Subtrochanteric diaphyseal	Good	1, primary fracture fixation	Hypertrophic	Mechanical, broken nail	Cephalomedullary nail	Blade plate 95°	Autograft + allograft	4	16
3	Female	19	None	Shaft diaphyseal	Good	1, primary fracture fixation	Oligotrophic	Mechanical	DCP plate	Intramedullary nail	Autograft + allograft	4	26
4	Female	54	Autoimmune hepatitis	Subtrochanteric diaphyseal (atypical fracture)	Moderate	1, primary fracture fixation	Atrophic	Biological, broken nail	Blade plate 95°	Blade plate 95°	Autograft + allograft	5	42
5	Female	71	DM, HTN	Shaft diaphyseal	Poor	2, primary fracture fixation and first nonunion operation	Atrophic	Biological broken nail	Intramedullary nail	Intramedullary nail	Autograft + allograft	6	36
6	Female	58	DM, HTN, DL, OSA, Asthma	Shaft diaphyseal	Good	2, primary fracture fixation and first nonunion operation	Atrophic	Biological	Distal femoral locked plate	Distal femoral locked plate	Autograft + allograft	5	30
7	Female	59	DM, HTN, DL, Vit D def, anemia, peripheral vascular disease post ipsilateral angioplasty	Supracondylar metaphyseal	Poor	1, primary fracture fixation	Atrophic	Biological	Distal femoral locked plate	Distal femoral locked plate	Autograft + allograft	9	44
8	Female	41	None	Shaft diaphyseal	Good	1, primary fracture fixation	Atrophic	Biological septic	Distal femur locked plate	Distal femur locked plate	Allograft	7	36

Abbreviations: DCP, dynamic compression plate; DM, diabetes mellitus; DL, dyslipidemia; HTN, hypertension; IHD, ischemic heart disease; NUSS, Non‐Union Scoring System; OSA, obstructive sleep apnea; Vit D def, vitamin D deficiency.

## 3. Discussion

The findings of this study are favoring the effectiveness of osteoperiosteal decortication with bone grafting for managing nonunion in different types of femoral fractures. Our study results are consistent with those of the most recent study conducted by the Judet group in 2008 [14], in which 99% of 297 patients achieved successful union within 8 months after undergoing decortication. Guyver et al.′s 2012 study [15], which investigated a group of 39 patients, further corroborated the positive outcome, demonstrating attainment of successful union in 92.3% of the surviving cases (36/39). This technique has gained recognition among other researchers [16–20] due to its consistent ability to yield satisfactory and effective outcomes.

The diamond concept emphasizes that successful fracture healing requires a balanced interaction between biological factors and mechanical stability, incorporating osteoinductive mediators, osteogenic cells, an osteoconductive scaffold, adequate vascularity, and optimization of host comorbidities [21]. Within this framework, the osteoperiosteal flap technique contributes primarily to the biological arm by preserving and stimulating osteoprogenitor cells and maintaining vascularized cortical bone chips, effectively creating an autologous “bone graft jacket” around the nonunion site that enhances local osteogenesis, whereas the chosen fixation provides the necessary mechanical environment for union.

Various factors, whether local or systemic, can increase the risk of nonunion. Treatment for nonunion can be conservative or operative, depending on the fracture site, type, and patient demographics, such as age, functional status, and activity. For active patients, osteoperiosteal decortication and bone grafting are recommended.

This technique has shown efficiency and efficacy in the treatment of nonunions. In his first published results in 1972, Judet [22] proposed that enhanced and expedited healing of pseudarthroses can be attained by encasing the fracture site with bone chips sourced from nonunited bone, provided these bone chips retain their vascular connection; this preserves their blood supply and stimulates osteogenesis by osteoprogenitor cells, ensuring improved consolidation and healing. In the context of our series, this procedure is aimed at achieving bone union and restore function by sequentially addressing both biological and mechanical factors. Decortication facilitates the formation of small vascularized cortical bone fragments acting as local autografts, whereas additional bone grafting fills defects or gaps at the nonunion site after decortication; these bone chips remain vascularized through the periosteum and surrounding muscles, thereby augmenting the local biological environment. The choice of internal fixation method is tailored to the fracture type in order to obtain a well‐reduced and, where appropriate, compressed nonunion site, thus providing a stable mechanical environment that complements the biological enhancement. In all our cases, the combined application of the decortication technique, bone grafting, and appropriately selected fixation directly contributed to successful healing of the nonunions.

We believe that the vascularity at the fracture site plays a major role in bone healing. This vascularity may be compromised by either the fracture itself or during primary fixation surgeries, in addition to any dormant comorbidity affecting the blood supply. With this technique, the affected site is surrounded by vascularized bone fragments and tissues that increase the healing rate.

Various methods have been used to predict the risk of nonunions in patients. The Calori [23] NUSS (Non‐Union Scoring System) generates a total score (multiplied by 2) that stratifies nonunions by complexity: scores of 0–25 indicate straightforward cases amenable to standard treatments; 26–50 suggest the need for specialized care; 51–75 warrant both specialized care and treatments; and scores exceeding 75 identify patients potentially suitable for primary amputation consideration.

This study has several key limitations. Due to the small sample size (*n* = 8) and lack of a comparison group in this descriptive case series, only basic descriptive statistics were reported, with no formal statistical analysis, confidence intervals, hypothesis testing, or comparative effectiveness assessment being feasible. A notable limitation of this investigation is the absence of documented functional reported outcomes for the patient cohort. This precludes a comprehensive assessment of the intervention′s impact on functional capacity and daily living activities. Additionally, the cohort demonstrated substantial heterogeneity across patient characteristics, nonunion morphologies, fixation methods (nails, locked plates, and blade plates), and grafting strategies (autologous iliac crest graft, allograft, or combined), which prevents definitive attribution of outcomes to any specific biological or mechanical intervention and underscores the need for larger, controlled prospective studies to validate these findings.

## 4. Conclusion

Osteoperiosteal decortication with bone grafting is a promising technique for treating aseptic nonunion of different types of femoral fractures. It has demonstrated good results for both hypertrophic and atrophic nonunions. The choice of bone graft used can vary depending on the size of the defect and availability of material in the institution. However, this technique is not advisable for treating articular fractures or areas with periosteal defects.

## Funding

No funding was received for this manuscript.

## Conflicts of Interest

The authors declare no conflicts of interest.

## Data Availability

The data that support the findings of this study are available from the corresponding author upon reasonable request.
